# Inhibition of miRNA-152-3p enhances diabetic wound repair via upregulation of PTEN

**DOI:** 10.18632/aging.103557

**Published:** 2020-07-03

**Authors:** Yan Xu, Tao Yu, Lei He, Liu Ouyang, Yanzhen Qu, Junjie Zhou, Yu Han, Deyu Duan

**Affiliations:** 1 Department of Orthopedics, Union Hospital, Tongji Medical College, Huazhong University of Science and Technology, Wuhan 430022, China; 2 Department of Orthopedic Surgery, Tongji Hospital, Tongji University School of Medicine, Shanghai 200065, China; 3 Department of Pathology, Union Hospital, Tongji Medical College, Huazhong University of Science and Technology, Wuhan 430022, China; 4 Department of Orthopedic Surgery, Shanghai Key Laboratory of Orthopedic Implants, Shanghai Ninth People’s Hospital, Shanghai Jiaotong University School of Medicine, Shanghai 200011, China

**Keywords:** microRNA-152-3p, diabetes, wound healing, PTEN

## Abstract

Diabetic foot ulcer (DFU) is a major complication of diabetes in the elderly population. The aim of this study was to investigate the potential mechanism of DFU at the molecular level and explore a feasible therapy for it. Using data from the Gene Expression Omnibus (GEO) database, we found that phosphatase and tensin homolog (PTEN) is differentially expressed between diabetic patients and those without diabetes. We also found that PTEN expression is regulated by glucose stimulation. In addition, decreased function of human umbilical vein endothelial cells (HUVECs) was found to be associated with reduction of PTEN. We identified microRNA-152-3p (miR-152-3p) to be a putative upstream negative regulator of PTEN, and in vivo and in vitro results indicated that miR-152-3p antagonist could restore HUVEC function and accelerate wound repair. Thus, miR-152-3p-induced downregulation of PTEN appears responsible for the delayed wound healing in DFU, and miR-152-3p inhibition may effectively accelerate wound repair, thereby providing a potential target for DFU therapy.

## INTRODUCTION

Diabetic foot ulcer (DFU) is a major complication of diabetes in the elderly population, bringing serious economic and mental burden to these patients [1]. It has been reported that angiogenesis is a key component of successful wound repair, and many factors affect angiogenesis [2]. A recent study showed that melatonin controls angiogenesis under different pathological and physiological conditions [3]. Human umbilical vein endothelial cells (HUVECs) are well known to aid in wound repair by regulating angiogenesis in the wound area [4]. Therefore, investigating the key genes related to angiogenesis and the angiogenesis-related mechanisms that are responsible for accelerated wound repair is important to find a potential treatment for patients with DFUs.

PTEN is a tumor suppressor, acting as a dual-specificity protein phosphatase, dephosphorylating tyrosine, serine, and threonine phosphorylated proteins. PTEN also acts as a lipid phosphatase, removing the phosphate in the D3 position of the inositol ring from phosphatidylinositol 3,4,5-trisphosphate, phosphatidylinositol 3,4-diphosphate, phosphatidylinositol 3-phosphate, and inositol 1,3,4,5-tetrakisphosphate with order of substrate preference in vitro [5]. PTEN is expressed in epithelial cells and activates several signaling cascades to effect the angiogenesis process [6]. Recent research found that PTEN plays a crucial role in successful wound repair through electric signals [7]. In this study, we found that PTEN expression is significantly downregulated in DFU patients, which is consistent with in vitro results of HUVECs stimulated by glucose. Thus, we speculated that PTEN could be a factor in the regulation of diabetic wound repair.

miRNAs are noncoding single-stranded RNAs with a short sequence that regulate gene expression. Their regulatory role in angiogenesis has been previously reported [8]. Recently, miR-21 was demonstrated to promote healing of cutaneous wounds in rats via activation of the MAPK/ERK signaling cascade [9]. The miR-26a/PTEN/Akt axis has also been shown to be associated with wound healing [10]. More attention has been recently devoted to the link between miRNAs and wound repair. However, the molecular mechanisms by which miRNAs influence target gene expression and thereby regulate diabetic wound repair remain incompletely understood and thus require further study.

In this study, we found that PTEN is differentially expressed between patients with diabetes and those without diabetes. In addition, in vitro and in vivo, our results indicate that PTEN upregulation promotes wound repair. miR-152-3p was demonstrated to be a valid upstream mechanism of PTEN, and inhibition of miR-152-3p was verified to promote wound repair, thus revealing a potential new avenue for treatment of DFU.

## RESULTS

### PTEN is a differentially expressed gene (DEG) in diabetic patients

**Figure 1:**
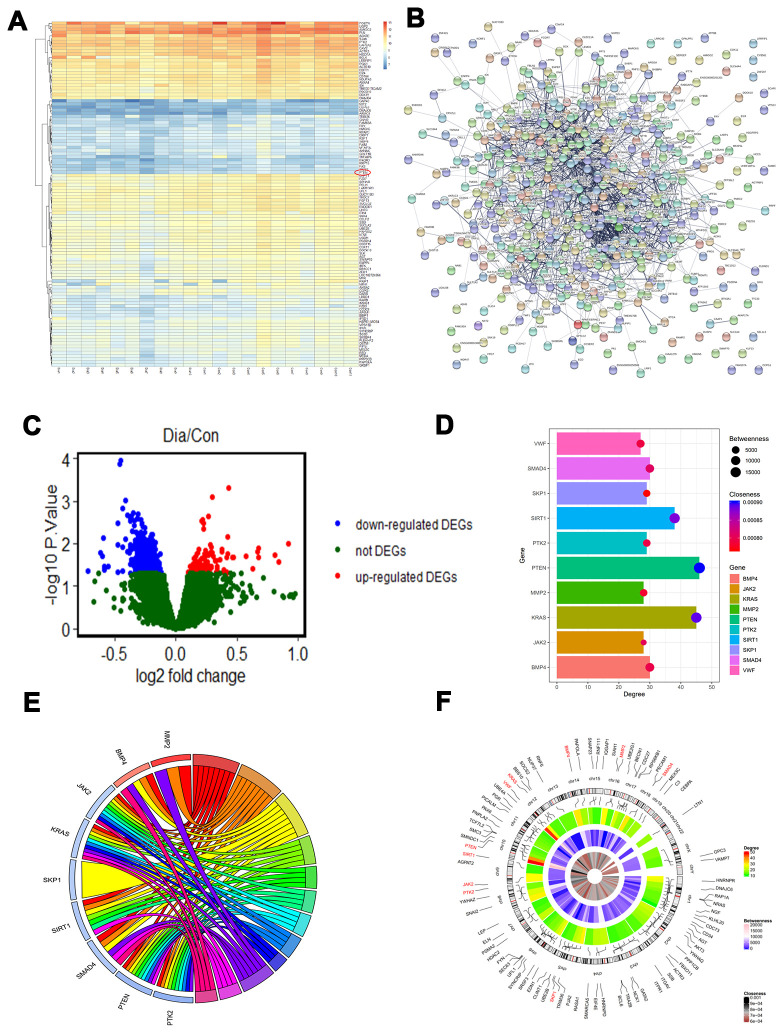
**PTEN is decreased in diabetic patients.** (**A**–**C**) Differentially expressed genes (DEGs) were identified between the diabetic patients and the controls. (**D**) The degree, betweenness, and closeness of the top 10 hub genes. (**E**) The degree centrality information of the top 50 genes from the DEG interaction network and their positions on chromosomes. (**F**) The results of enrichment analysis of hub genes.

The GSE13760 data set based on the Affymetrix Human Genome U133A Array (GPL571) platform was retrieved from National Center for Biotechnology Information (NCBI) Gene Expression Omnibus (GEO) and included 10 patients with type 2 diabetes and 11 control subjects. The hierarchical clustering analysis of all DEGs (log2 fold change) is shown in the heatmap in Figure 1A. Upregulated and downregulated DEGs were identified (Figure 1B–1C). The network of protein protein interaction (PPI) was first constructed using STRING (https://string-db.org/). The top 10 hub genes with the highest degrees were then identified using Cytoscape software. These genes were PTEN, KRAS, SIRT1, SMAD4, BMP4, SKP1, PTK2, MMP2, JAK2, and VWF (Table 1). We performed functional and pathway enrichment analysis of the genes in the module. The top three terms were selected according to *P* value when more than three enriched terms were identified in each category (Table 2). The degree, betweenness, and closeness of each hub gene are shown in Figure 1D and 1E. Genes involved in the top three modules with the highest MCODE score were imported into DAVID (https://david.ncifcrf.gov/) for enrichment analysis. Under the term of biological process, DEGs were significantly enriched in cell adhesion, biological adhesion, and cell-cell adhesion. Under the term of molecular functions, DEGs were significantly enriched in receptor binding, protein complex, and intermediate filament binding. Results of enrichment analysis of hub genes are shown in Figure 1F.

**Table 1:** Degree of top 10 genes in the network.

**Gene ID**	**Gene name**	**Degree**	**Betweenness**	**Closeness**
PTEN	Phosphatase and tensin homolog	46	19692	9.01E-4
KRAS	KRAS Proto-Oncogene, GTPase	45	16019	8.90E-4
SIRT1	Sirtuin 1	38	15344	8.80E-4
SMAD4	SMAD Family Member 4	30	7456	8.10E-4
BMP4	Bone Morphogenetic Protein 4	30	8573	7.97E-4
SKP1	S-Phase Kinase Associated Protein 1	29	4211	7.66E-4
PTK2	Protein Tyrosine Kinase 2	29	4815	7.95E-4
MMP2	Matrix Metallopeptidase 2	28	4694	7.84E-4
JAK2	Janus Kinase 2	28	3061	8.05E-4
VWF	Von Willebrand Factor	27	6133	7.93E-4

**Table 2:** Functional and pathway enrichment analysis of the genes in the module.

A, Biological processes				
Term	Name	Count	P-value	Genes
GO:0008284	Positive regulation of cell proliferation	8	2.9E-8	BMP4, PTK2, KRAS, SMAD4, JAK2, PTEN, SIRT1, MMP2
GO:0007167	Enzyme linked receptor protein signaling pathway	8	6.5E-8	BMP4, PTK2, KRAS, SMAD4, JAK2, PTEN, SIRT1, MMP2
GO:0031401	Positive regulation of protein modification process	8	2.2E-7	BMP4, PTK2, KRAS, SMAD4, JAK2, SKP1, PTEN, SIRT1
B, Cellular component				
Term	Name	Count	P-value	Genes
GO:0009898	Cytoplasmic side of plasma membrane	4	1.1E-4	PTK2, KRAS, JAK2, PTEN
GO:0098562	Cytoplasmic side of membrane	4	1.4E-4	PTK2, KRAS, JAK2, PTEN
GO:0031234	Extrinsic component of cytoplasmic side of plasma membrane	3	1.7E-3	PTK2, KRAS, JAK2
C, Molecular functions				
Term	Name	Count	P-value	Genes
GO:0019904	Protein domain specific binding	5	2.6E-4	PTK2, KRAS, JAK2, PTEN, SIRT1
GO:0019901	Protein kinase binding	4	2.9E-3	PTK2, JAK2, PTEN, SIRT1
GO:0019900	Kinase binding	4	4.0E-3	PTK2, JAK2, PTEN, SIRT1
D, KEGG pathway				
Term	Name	Count	P-value	Genes
hsa05200	Pathways in cancer	6	6.2E-5	BMP4, PTK2, KRAS, SMAD4, PTEN, MMP2
hsa04068	FoxO signaling pathway	4	5.6E-4	KRAS, SMAD4, PTEN, SIRT1
hsa04550	Signaling pathways regulating pluripotency of stem cells	4	6.3E-4	BMP4, KRAS, SMAD4, JAK2
KEGG, Kyoto Encyclopedia of Genes and Genomes.				

Top 3 terms were selected according to P-value when more than 3 terms enriched terms were identified in each category.

### PTEN level fluctuates with glucose stimulation

**Figure 2:**
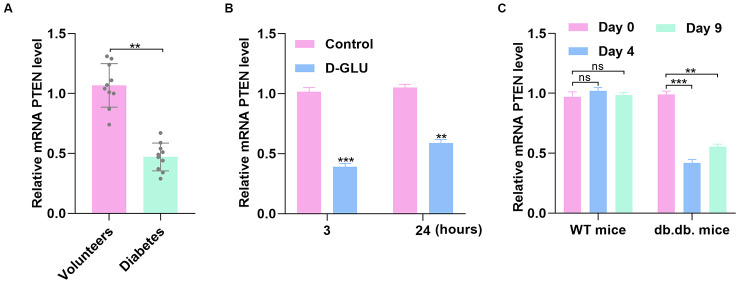
**PTEN expression fluctuates with simulation by glucose.** (**A**) The PTEN level was lower in DFU patients than in nondiabetic controls as measured by qRT-PCR analysis. (**B**) PTEN level was determined via qRT-PCR in HUVECs treated with D-glucose. (**C**) PTEN expression was significantly decreased 3 and 7 days after wounding in diabetic mice compared with nondiabetic mice. Data are the mean ± SD of three independent experiments. *P <0.05, **P <0.01, ***P <0.001.

Because the informatics results indicated significantly different expression of PTEN between diabetic patients and healthy controls, we collected serum samples from patients with DFU and the controls to investigate the PTEN level between the two groups. Quantitative real-time polymerase chain reaction (qRT-PCR) analysis demonstrated that PTEN level was significantly reduced in the DFU group compared with controls (Figure 2A). To investigate whether PTEN level in HUVECs is affected by diabetic stimuli, PTEN expression was measured in HUVECs treated with phosphate-buffered saline (PBS) or D-glucose via qRT-PCR analysis. The results indicated that PTEN level was decreased in HUVECs stimulated with D-glucose at 3 and 24 hours (Figure 2B). Similarly, PTEN expression was significantly decreased at 4 and 9 days after wounding in diabetic mice, which was not be found in nondiabetic mice (Figure 2C).

### Reduction of PTEN hindered wound repair in vivo

**Figure 3:**
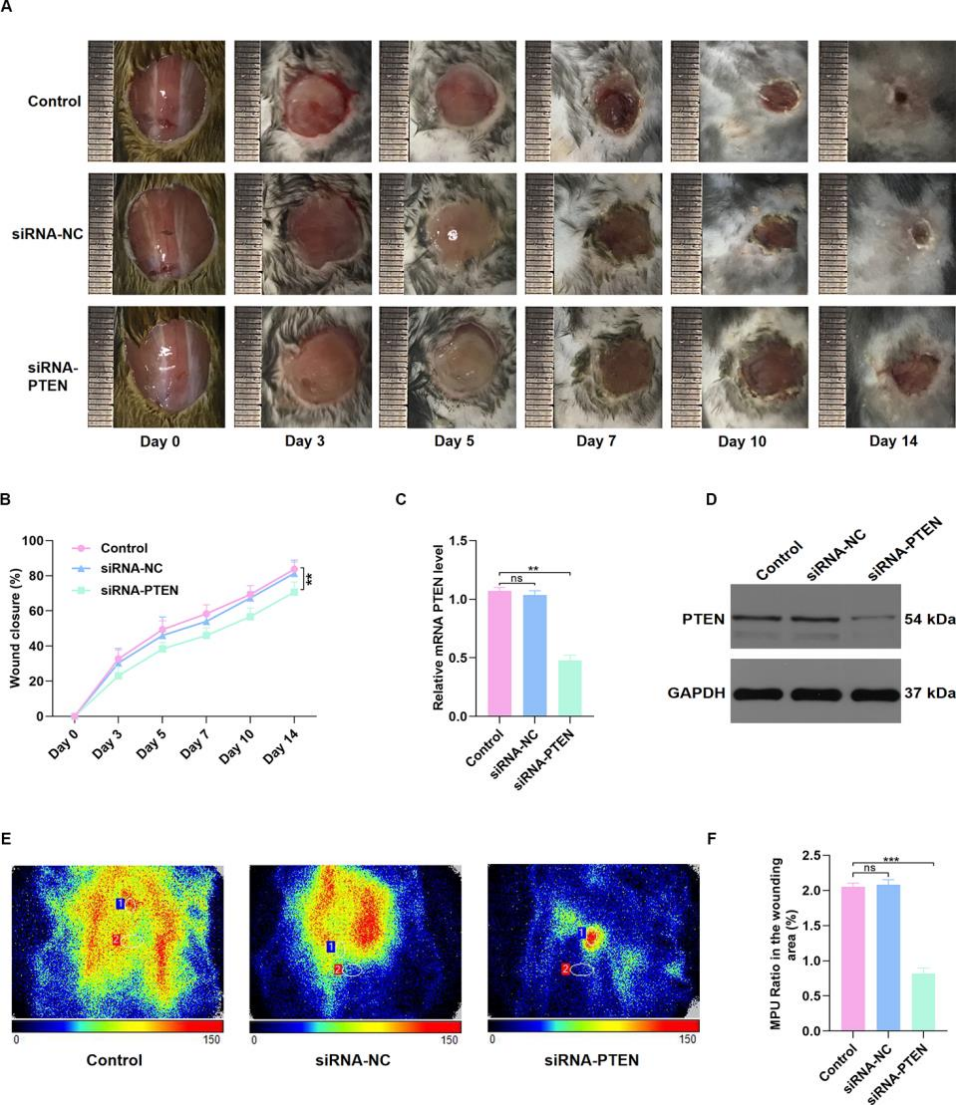
**PTEN inhibition delayed wound healing in vivo.** (**A**–**B**) The general view of wound repair in the mice models. (**C**–**D**) PTEN expression of skin tissue in the wound area was measured by qRT-PCR and WB analyses. (**E**–**F**) Doppler results of blood perfusion in the wound area among the three groups. Data are the mean ± SD of three independent experiments. *P <0.05, **P <0.01, ***P <0.001.

Next, C57BL/6 mice with full-thickness back wounds were locally injected with PBS, siRNA-NC, or siRNA-PTEN at days 0, 3, 5, 7, 10, and 14 after injury. The results showed that the repair process was significantly sustained by local siRNA-PTEN injection (Figure 3A–3B). At day 14 after injury, the skin tissues of the wound were collected to determine the PTEN level, and qRT-PCR and western blot (WB) results indicated that PTEN level was significantly decreased in the siRNA-PTEN group (Figure 3C–3D). Interestingly, Doppler detection found a weakened local mean perfusion unit (MPU) ratio consistent with the ability of this siRNA to delay wound repair (Figure 3E–3F).

### PTEN regulated HUVEC function in vitro

**Figure 4:**
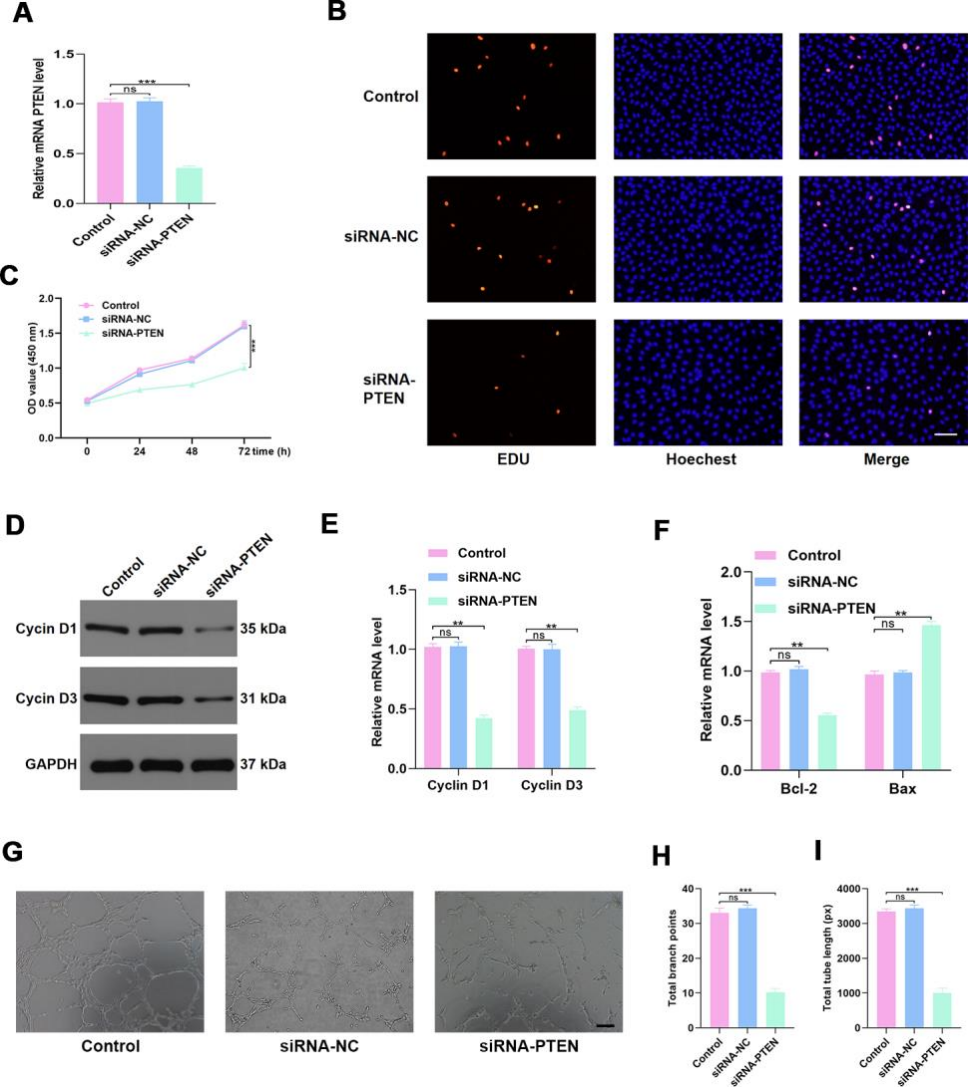
**PTEN regulates HUVEC function.** (**A**) PTEN expression was measured by qRT-PCR in the different treated groups. (**B**–**C**) Effect of diabetic exosomes on HUVEC proliferation measured by CCK8 and EDU incorporation assays. Scale bar: 100 μm. (**D**–**E**) The effect of PTEN on the levels of the proliferation-related proteins Cyclin D1 and Cyclin D3, assessed by WB and qRT-PCR analysis. (**F**) The effect of PTEN on the levels of the apoptosis-related proteins Bax and Bcl-2. (**G**–**I**) Effects of PTEN on tube formation ability of HUVECs. Scale bar: 200 μm. Data are the mean ± SD of three independent experiments. *P <0.05, **P <0.01, ***P <0.001.

To investigate the effect of PTEN on HUVEC function, the siRNA-PTEN was constructed, and the qRT-PCR result confirmed the efficacy of this siRNA in knocking down the expression of PTEN (Figure 4A). To detect the proliferation of HUVECs in the different treatment groups, the EdU cell proliferation assay and the cell counting kit-8 (CCK8) proliferation assay were performed. The results indicated that proliferation of HUVECs was sustained following siRNA-PTEN treatment (Figure 4B–4C). Similarly, the proliferation-related genes Cyclin D1 and Cyclin D3 were found to be decreased in HUVECs treated with siRNA-PTEN (Figure 4D–4E). To investigate the effect of PTEN on the apoptosis of HUVECs, qRT-PCR was used to measure apoptosis-related genes levels and indicated a significant reduction of Bcl-2 expression and increasing Bax expression in the siRNA-PTEN group (Figure 4F). Moreover, the tube formation assay was performed to explore angiogenesis in the different groups and indicated a significant reduction of tube formation in the siRNA-PTEN group (Figure 4G–4I).

### miR-152-3p is a potential upstream mechanism of PTEN

**Figure 5:**
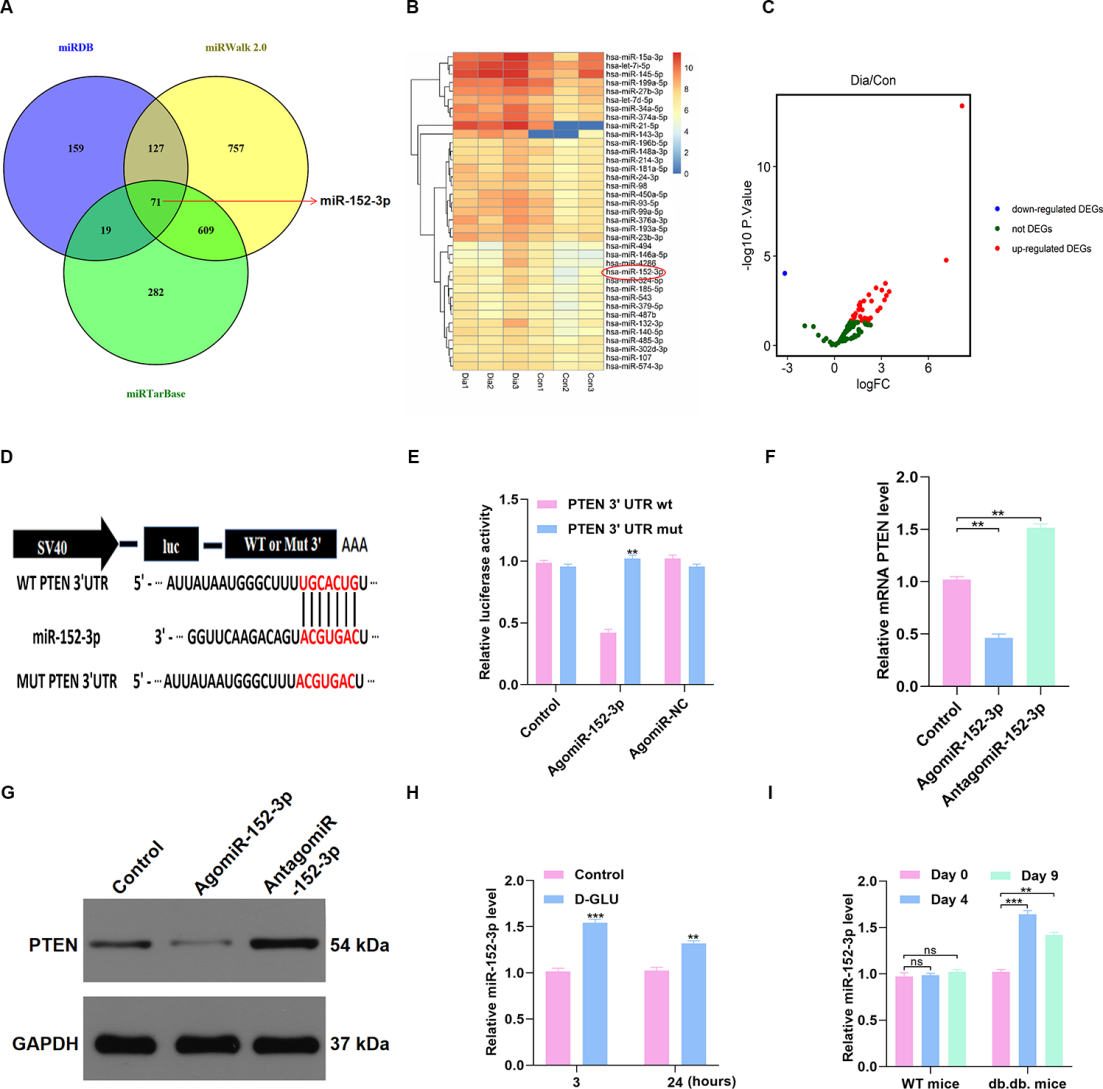
**miR-152-3p acts as a potential upstream mechanism of PTEN.** (**A**–**C**) The potential upstream miRNAs of PTEN were identified using online predicting tools and bioinformatics analysis. (**D**–**E**) Results of luciferase assays for miR-152-3p and PTEN. (**F**–**G**) The PTEN level in the different groups was measured by qRT-PCR and WB analyses. (**H**) miR-152-3p level was determined in HUVECs treated with D-glucose via qRT-PCR analysis. (**I**) miR-152-3p expression was significantly increased at 3 and 7 days after wounding in diabetic mice compared with nondiabetic mice. Data are the mean ± SD of the three independent experiments. *P <0.05, **P <0.01, ***P <0.001.

The underlying upstream mechanism of PTEN was then investigated using online predicting tools, including miRTarBase (http://mirtarbase.mbc.nctu.edu.tw/php/index.php), miRDB (http://mirdb.org/), and miRWalk (http://zmf.umm.uni-heidelberg.de/apps/zmf/mirwalk2/). The results showed that miR-152-3p is a potential upstream miRNA (Figure 5A). The GSE84971 data set from the GEO database was used to identify the differentially expressed miRNAs between DFU patients and controls, and miR-152-3p was found to be a key differentially expressed miRNA (Figure 5B–5C). Thus, the luciferase assay was carried out to verify the association between miR-152-3p and PTEN. We found that miR-152-3p could specifically bind to the predicted target region of the PTEN mRNA (Figure 5D–5E). In addition, miR-152-3p overexpression resulted in suppression of PTEN expression in HUVECs (Figure 5F–5G). Moreover, we found that miR-152-3p expression was increased by D-glucose stimulation at 3 and 24 hours (Figure 5H). Similarly, miR-152-3p expression was significantly increased at 4 and 9 days after surgery in diabetic mice; this was not seen in nondiabetic mice (Figure 5I).

### miR-152-3p regulated HUVEC function in vitro

**Figure 6:**
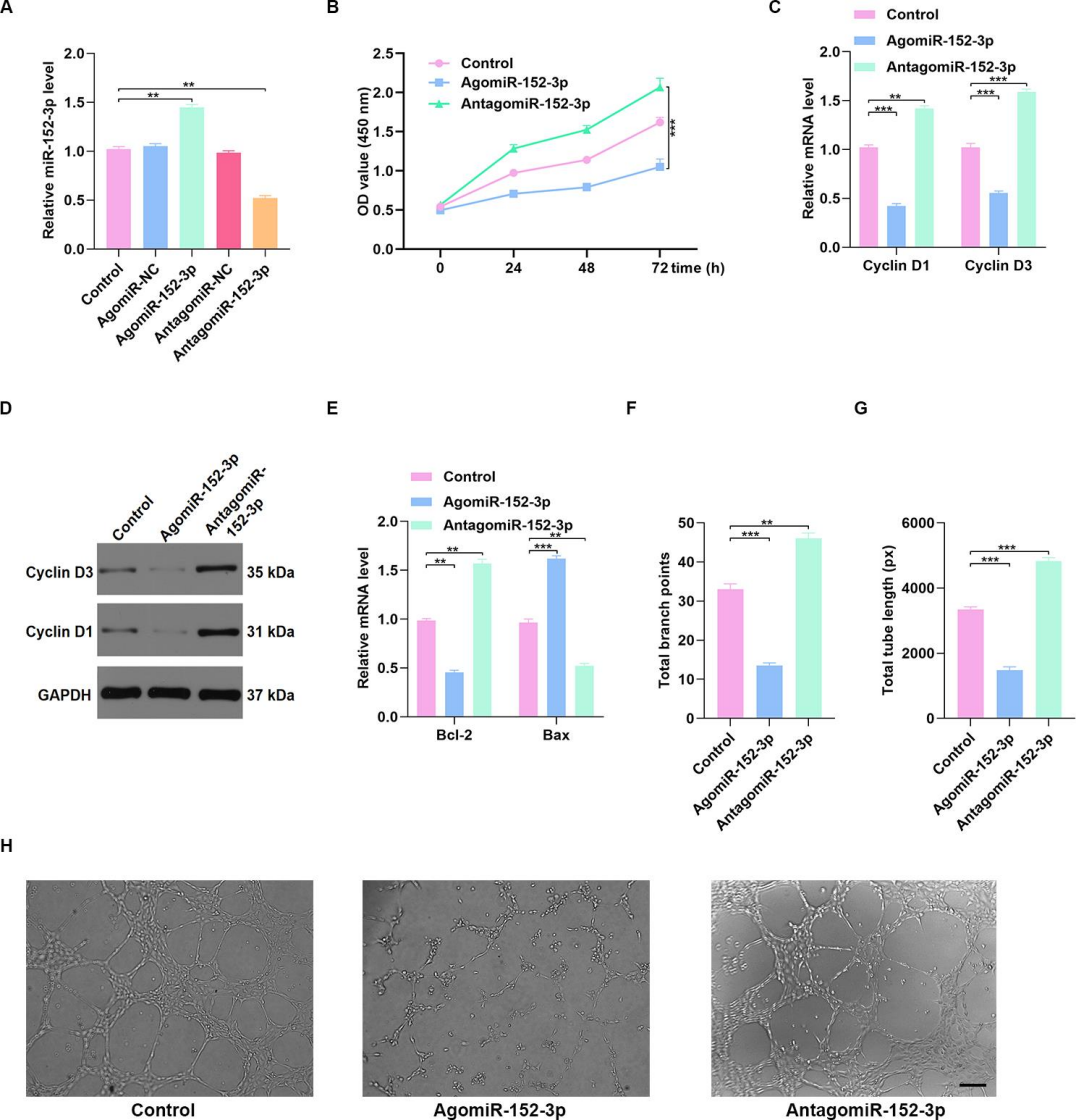
**Inhibition of miR-152-3p enhanced HUVEC function.** (**A**) The qRT-PCR result confirmed the effect of miR-152-3p agonist and antagonist on the expression of miR-152-3p. (**B**) CCK8 proliferation assay was performed, and the results demonstrated that proliferation of HUVECs was increased after miR-152-3p antagonist treatment. (**C**–**D**) The proliferation-related mRNAs in the different groups were measured using qRT-PCR and WB analyses. (**E**) The apoptosis-related mRNAs were assessed using qRT-PCR analysis. (**F**–**H**) Effects of miR-152-3p on the tube formation ability of HUVECs. Scale bar: 200 μm. Data are the mean ± SD of three independent experiments. *P <0.05, **P <0.01, ***P <0.001.

To investigate the effect of miR-152-3p on HUVEC function, miR-152-3p agonist (agomiR-152-3p) and miR-152-3p antagonist (antagomiR-152-3p) were constructed. The qRT-PCR results confirmed that miR-152-3p agonist and antagonist affect the expression of miR-152-3p (Figure 6A). CCK8 proliferation assay results indicated enhanced proliferation of HUVECs after antagomiR-152-3p treatment (Figure 6B). In addition, the qRT-PCR and WB results indicated that antagomiR-152-3p induces HUVEC proliferation and suppresses apoptosis (Figure 6C–6E). The effect of antagomiR-152-3p on angiogenesis in HUVECs was also measured, using the tube formation assay; results indicated a significant enhancement of tube formation in HUVECs after antagomiR-152-3p treatment (Figure 6F–6H).

## DISCUSSION

Recently, the underlying mechanisms of a variety of diseases have been analyzed using microarray methods, which has led to functional genomic studies [11, 12]. Similarly, microarrays have been used to determine the key genes related to diabetes-associated impaired wound healing and have identified a range of relevant genes including the wnt family member 9B (Wnt9B), β-catenin, and transforming growth factor-β (TGF-β) [13–15]. These DEGs are closely related to the changed expression of genes that have crucial structural and functional roles in this context, leading to their modulation of diabetic wound progression. In this study, we identified PTEN as a significant factor in diabetes-related impaired wound healing using microarray data sets with Gene Ontology (GO) and Kyoto Encyclopedia of Genes and Genomes (KEGG) pathway enrichment analyses.

Exosome is derived from cells and released into the circulation, and functional exosomal miRNAs are delivered to target cells. However, the molecular mechanisms that regulate this process, including the tissue of origin of the exosomal miRNAs, the specific target tissues of exosomal miRNAs, and the manner in which the miRNAs are delivered, remain elusive. miRNAs are noncoding single-stranded RNAs with a short sequence that regulate gene expression. A variety of miRNAs have been reported to be associated with wound repair. Recently, for example, miR-199a-3p was demonstrated to delay wound healing by sustaining cell proliferation [16]. To search for the potential upstream miRNAs of PTEN, we used online predicting tools and microarray data sets. After rigorous screening, 71 miRNAs, including miR-152-3p, were identified as candidates. Using the GSE84971 data set from the GEO database, we found that miR-152-3p was significantly differentially expressed between DFU patients and controls. Furthermore, after reviewing related research, we found that miR-152-3p is associated with wound repair. Interestingly, a recent study reported a close association between miR-152-3p and PTEN and regulation of apoptosis in progression of heart failure [17]. Therefore, we investigated the relationship between miR-152-3p and PTEN.

miRNAs have been demonstrated to regulate gene expression in the target cells in which they are internalized [18]. Given that the miRNAs circulate in the blood, the cells of the vascular endothelium are likely to be highly exposed to these micromolecules, making them susceptible to their regulatory roles. Abnormal function of endothelial cells is a crucial hallmark of diabetes and leads to a variety of undesirable consequences [19]. In our study, overexpression of miR-152-3p was found in DFU patients and had harmful effects on the vascular function of HUVECs. When agomiR-152-3p was transferred into HUVECs, their proliferation and angiogenesis were significantly impaired, whereas the rate of apoptosis increased significantly.

PTEN was demonstrated to be expressed in epithelial cells and to activate several signaling cascades that affect angiogenesis [20]. Angiogenesis is major contributor to blood perfusion in wounds, which brings oxygen and nutrients to the wound and thus accelerates the repair process [21]. Delayed and weakened angiogenesis is a crucial factor in the delayed healing that characterizes DFU [22]. PTEN has been reported to be involved in a variety of in vivo biological processes and affects angiogenesis by regulating endothelial cell proliferation, apoptosis, and migration [23]. In this study, low PTEN expression was found in diabetic patients, which is consistent with the finding of low PTEN expression in HUVECs after D-glucose stimulation. Thus, we speculate that PTEN expression affects wound repair in diabetic patients.

In conclusion, we found that PTEN expression is reduced in patients with diabetes, which is regulated by the expression of miR-152-3p. The circulating miR-152-3p and its damaging effects on HUVECs are probably main contributors to angiopathy in patients with DFU. Thus, miR-152-3p inhibition may provide a potential therapeutic target for the treatment of DFU.

## MATERIALS AND METHODS

### Ethical consent

From August 2016 to October 2018, peripheral blood samples (10 healthy volunteers and 10 DFU patients) were collected from patients at the Union Hospital, Tongji Medical College, Huazhong University of Science and Technology for measurement of PTEN and miR-152-3p levels. The patient and animal studies were approved by the Committee of Clinical Ethics in the Tongji Medical College, Huazhong University of Science and Technology, and informed consent was obtained from all participants.

### Animal wound model

Male C57BL/6J mice (6-7 weeks old, 25-30 g) and male db/db mice (BKS.Cg-Dock7m +/+ Leprdb/J strain) were donated by the Center of Experimental Animal, Tongji Medical College, Huazhong University of Science and Technology. Mice were single-caged and housed at room temperatures of 18°C with a 12/12-hour light-dark cycle. The mice had free access to water and were fed a chow diet. After anesthetizing using pentobarbital sodium (50 mg/kg), mice were shaved by a depilatory cream, and a full-thickness cutaneous skin wound (10 mm in diameter) was produced using punch biopsy needle. Mice were then randomly divided into several groups based on the different treatments. Briefly, the mice were subcutaneously injected with samples around the wounds at four injection sites (25 μL per site) on days 0, 3, 5, 7, 9, and 11 after wounding (n = 6). At days 0, 3, 5, 7, 10, and 14 after wounding, the wounds were photographed and measured with a caliper rule.

### Wound closure rate and Doppler detection

ImageJ software (National Institutes of Health) was used to assess the wound closure rate at days 0, 3, 5, 7, 10, and 14 after surgery. In addition, laser speckle contrast imaging (LSCI) was performed to evaluate the condition of blood perfusion in the wound area at day 10 after surgery. Doppler images were captured using the same scan area dimensions at a constant distance from the wound surface. PIMSoft (Moor Instruments Ltd, Axminster, UK) was used, and flux images of each wound site were analyzed to determine the mean perfusion unit (MPU) ratio, which was calculated by comparing the MPU in the wound area (ROI-1) with the area around the wound (ROI-2).

### HUVEC culture

Cells were purchased from the Cell Bank of the Chinese Academy of Science, Shanghai, China. RPMI 1640 (ThermoFisher Scientific, MA, USA) containing 10% FBS (Gibco, NY, USA) was used to culture the HUVECs. Cells were cultured at 37°C with 5% CO_2_ and 95% humidity. The Lipofectamine 3000 (ThermoFisher Scientific) was used for cell transfection. For the agomiR and antagomiR transfection steps, constructs from GenePharma (Shanghai) were used, and transfection was performed at a concentration of 200 mM. The PTEN-specific siRNA (Ribobio, Guangzhou, China) was transfected at a concentration of 50 mM.

### CCK8 proliferation assay

Briefly, cells (5 × 10^3^) were added into the 96-well plates and cultured for 24, 48, or 72 hours. CCK8 reagent (#96992, Sigma-Aldrich, MO, USA) was then added to the cells in serum-free medium for 2 hours, followed by measurements of absorbance at 450 nm.

### Tube formation assay

Sixty microliters of cold Matrigel (#354234, Corning, NY, USA) were put into each well and incubated at 37°C for 30 minutes. HUVECs, 2.5 × 10^4^ per well, were then added to the Matrigel-coated 96-well plate and randomly assigned to different groups according to the different treatments. After 8 hours of culture, three random fields of view were captured with an inverted microscope. Tube length and total branch points were quantified using ImageJ software.

### Luciferase reporter assay

Position 2254-2260 of 3’UTR of PTEN mRNA containing the putative target site of miR-152-3p was determined using an online predicting tool (TargetScan 7.1) and amplified by PCR from the cDNA of HUVECs and ligated into the pGL3-basic vector (Promega Corporation). pGL3-*PTEN*-3’UTR-mutant (Mut) was created by introducing two site mutations into miR-152-3p potential target sites using Quick ChangeSite-Directed Mutagenesis kits (Agilent Technologies, Inc.). pGL3-*PTEN*-3’UTR-wild-type (W; 200 ng) or pGL3-*PTEN*-3’UTR-Mut (200 ng) was co-infected with *Renilla* plasmid into HUVECs using Lipofectamine 3000 (ThermoFisher Scientific). This was followed by transfection of miR-NC mimic (10 nM) or miR-152-3p mimic (10 nM) for 48 hours at 37°C. The miR-NC mimic and miR-152-3p mimic transfection kits were supplied by Shanghai GenePharma Co., Ltd. The Dual-Luciferase Reporter assay system (Promega Corporation) was used to measure the relative luciferase activity of each well. The firefly luciferase expression was normalized to *Renilla*.

### qRT-PCR

TRIzol reagent (ThermoFisher Scientific) was used to isolate total RNA from cell and tissue samples. The purified RNA was then reverse transcribed into cDNA using the ReverTra Ace qPCR RT Master Mix (Toyobo Life Science), according to the manufacturer’s protocol. RT reaction was conducted for 15 minutes at 42°C, followed by 5 minutes at 98°C, and the reaction volume was 20 μL. The qPCR thermocycling conditions were as follows: initial denaturation at 95°C for 30 seconds, 40 cycles at 95°C for 5 seconds and 60°C for 30 seconds, and the reaction volume was 25 μL. GAPDH served as an internal control. Relative miRNA expression levels were normalized to those of the internal control (GAPDH) and were calculated according to the 2^-ΔΔCt^ method. All experiments were conducted in triplicate, and the primer sequences were as follows: miR-152-3p, forward, ACACTCCAGCTGGGTCAGTGCATGACAG, reverse, CTCAACTGGTGTCGTGGAGTCGGCAATTCAGTTGAGCCAAGTT; Bcl-2, forward, GATAACGGAGGCTGGATGC, reverse, TCACTTGTGGCCCAGATAGG; Bax, forward, CCCTTTTGCTTCAGGGTTTC, reverse, GAGACACTCGCTCAGCTTCTTG; Cyclin D1, forward, TTGCCCTCTGTGCCACAGAT, reverse, TCAGGTTCAGGCCTTGCACT; Cyclin D3, forward, CTGGCCATGAACTACCTGGA, reverse, CCAGCAAATCATGTGCAATC; PTEN, forward, TTTGAAGACCATAACCCACCAC, reverse, ATTACACCAGTTCGTCCCTTTC; GAPDH, forward, CCGTTGAATTTGCCGTGA, reverse, TGATGACCCTTTTGGCTCCC.

### Western blot

The cells were washed three times with PBS, and radioimmunoprecipitation assay lysis buffer (Aspen Pharmacare Holdings Ltd.; cat. no. AS1004) was used to extract the total proteins from cells. Cell lysates (1 × 10^4^) were subjected to 10% SDS-PAGE followed by determination of protein concentration by the bicinchoninic acid method. The proteins (50 μg) were then transferred onto a 10% SDS-PVDF membrane. The PVDF membrane was blocked by 5% bovine serum albumin (Abcam) at room temperature for 2 hours. A chemiluminescence detection system (Canon, Inc.; cat. no. LiDE110) was then used to visualize proteins based on the provided instructions. Antibodies used were as follows: anti-PTEN (1:1000; Abcam; cat. no. Ab32199), anti-Cyclin D1 (1:500; Abcam; cat. no. Ab134175), anti-Cyclin D3 (1:500; Abcam; cat. no. Ab112034), anti-Bcl-2 (1:500; Abcam; cat. no. Ab185002), anti-Bax (1:500; Abcam; cat. no. Ab182734), and anti-GAPDH (1:10,000; Abcam; cat no. ab37168). All experiments were conducted in triplicate.

### Microarray data and bioinformatics analysis

Microarray data of six diabetic patients and three normal controls were retrieved from a previously performed microRNA expression assay publicly deposited in the NCBI Gene Expression Omnibus (GEO, http://www.ncbi.nlm.nih.gov/geo). Differential expression identification was performed by GEO2R inbuilt function with default setting (https://www.ncbi.nlm.nih.gov/geo/geo2r/). *P* values <0.05 were considered as statistically significant for DEGs. Log2 mRNA gene expression was visualized by heatmap using R package pheatmap. DEGs were imported into Search Tool for the Retrieval of Interacting Genes (STRING) to construct the PPI network. Then the TSV file of PPI network was imported into Cytoscape 3.7.2. The degree, betweenness, and closeness of the genes were calculated using the plugin software Centiscape 2.2 in Cytoscape. Database for Annotation, Visualization, and Integrated Discovery (DAVID), an online bioinformatics resource, was used to perform GO and KEGG pathway enrichment analysis. The top three GO terms in biological process, molecular function, and cellular component or KEGG pathways were obtained using the enrichment analysis. The top 10 genes with the highest degrees were visualized with ggplot2 and were considered as hub genes. The result of enrichment analysis of hub genes was visualized with GOplot. The circular visualization of chromosomal positions and connectivity of degree of the top 50 genes was achieved with Circular Visualization in R.

### Statistical analysis

The data are presented as the means±SD. Student’s t-tests were performed to compare the two groups, and more than two groups were compared using one-way ANOVAs with Tukey post hoc. Analyses were conducted using Graphprism 8.0. *P* <0.05 was the significance threshold.

### Ethical approval

Ethics Committee of Union Hospital, Tongji Medical College, Huazhong University of Science and Technology.
